# Coprological Detection of Gastrointestinal Nematodes in Intensively Managed Poultry Farms in Bishoftu, Ethiopia

**DOI:** 10.1155/japr/9138062

**Published:** 2025-09-11

**Authors:** Misgana Tefera, Maraki Wasiyhun

**Affiliations:** Department of Veterinary Pathology and Parasitology, College of Veterinary Medicine and Agriculture, Addis Ababa University, Addis Ababa, Ethiopia

**Keywords:** commercial farm, nematode infections, poultry, risk factors

## Abstract

The poultry industry is an infant but rapidly growing sector in Ethiopia. Although poultry farming is one of the Ethiopian government's developmental initiatives, the sector is facing various challenges, particularly due to infectious diseases. Among infectious diseases, helminthiasis is one of the challenges affecting poultry production. A cross-sectional study was conducted from April to June 2023 to determine the occurrence and associated risk factors of nematode infections in intensively managed commercial poultry farming in Bishoftu, Ethiopia. Representative pooled fecal samples were collected from 60 poultry farms and examined for the presence of worm eggs by using the flotation technique. Gastrointestinal nematode eggs were identified based on their morphological characteristics. Coprological analysis results reveal that out of 60 poultry farms screened, 19 (31.7%) were tested positive. The most identified worms were *Ascaridia galli* 11 (18.3%), followed by *Heterakis gallinarum* 3 (5%), *Trichostrongylus tenuis* 3 (5%), *Syngamus trachea* 2 (3.3%), and *Capillaria* species 1 (1.7%). Production types, management practices, and proximity to other farms were found to significantly (*p* < 0.05) influence the occurrence of worm infections. The prevalence of worm infections was significantly lower (6.06%, *p* < 0.05) in the farms using footbaths as compared to farms not utilizing footbaths (62.96%). Similarly, significantly higher (*p* < 0.05) prevalence was observed in the farms that did not apply wet cleaning (76.2%) and chemical disinfection (66.7%) as compared to those using wet cleaning (7.7%) or chemical disinfection (16.7%) during farm downtime. However, there was no significant difference (*p* > 0.05) between age groups and poultry farm scale. This study strongly suggests that gastrointestinal nematode infections pose a significant challenge to poultry production. Therefore, implementing effective worm control strategies, such as regular deworming, implementing proper farm hygiene practices, and strict biosecurity measures, is strongly recommended.

## 1. Introduction

The global poultry population was estimated to be 26 billion in 2022 [1]. Poultry farming has grown tremendously in recent years and has become one of the most intensive types of animal husbandry [2]. In developing countries such as Ethiopia, poultry production has a significant role in the economic, social, nutritional, and cultural benefits. It provides high animal protein in the form of eggs and meat [3, 4]. However, the contribution of Ethiopia's poultry sector to the national economy remains limited due to various constraints, including poor husbandry practices, inadequate feed quality, and the incidence and widespread distribution of infectious and noninfectious diseases [5, 6]. In Ethiopia, various poultry diseases have been reported, particularly in the commercial breeds raised in intensive farming systems. Common reported diseases include Newcastle disease, salmonellosis, chronic respiratory disease (CRD), and parasitic infections, all of which pose major challenges to poultry production [7].

Among these diseases, internal parasites such as cestodes, nematodes, and *Protozoa* (particularly *Eimeria* species) are the most important gastrointestinal (GI) parasites known to reduce poultry's productivity under different management systems. These parasites cause poor growth, decreased egg production, malnutrition, decreased feed conversion ratio, decreased disease resistance, aggravating existing disease conditions, and death in chickens [8, 9]. Nematodes are among the parasitic infectious agents causing a serious challenge in terms of economic losses by affecting the production performance of chickens and increasing treatment costs [5, 6]. Common nematode parasites affecting chickens are *Ascaridia galli*, *Heterakis gallinarum*, *Syngamus trachea*, *Trichostrongylus tenuis*, and *Capillaria* species [7, 10]. These worms are widely distributed globally [10, 11], and the frequency and severity of infections are influenced by factors such as age, breed, and other host and environmental variables [12].

Most existing research studies conducted in Ethiopia have predominantly focused on helminth infections in backyard or village poultry production systems, with limited research data available in commercial poultry farms, where management practices differ substantially from commercial intensive production systems. Even though the impact of GI nematode infection on poultry health and productivity is well recognized, a significant research gap remains regarding their prevalence and associated risk factors, particularly in intensive commercial poultry production systems. Factors such as biosecurity measures, flock size, hygiene practices, housing design, and proximity between farms may commonly influence nematode transmission dynamics in intensive farming. Hence, in order to design effective and evidence-based interventions and control strategies, it is very much essential to know the occurrence and associated risk factors of worm infections in intensive commercial poultry farms. Therefore, this study is aimed at determining the occurrence and risk factors associated with nematode infections in chickens raised under intensive commercial production farming systems in Bishoftu town.

## 2. Material and Methods

### 2.1. Study Area

**Figure 1:**
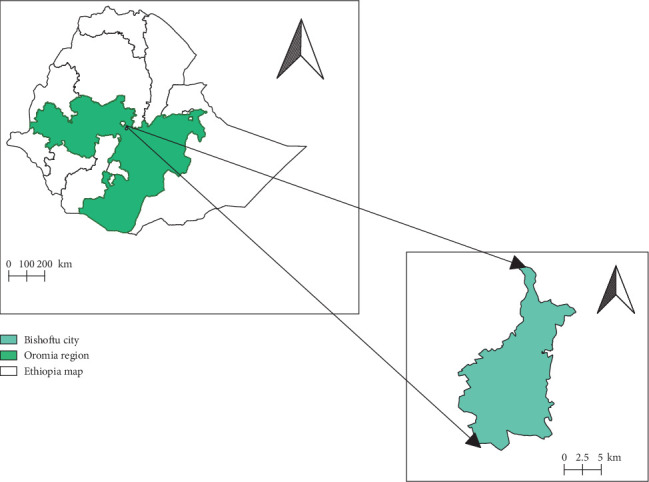
Geographical location of the study area.

The study was conducted from April to June 2023 in Bishoftu town, located in the East Shewa zone of the Oromia region state. Bishoftu is located 47 km southeast of the capital city of Ethiopia, Addis Ababa (Figure 1). It is situated at 9° N latitude and 40° E longitude. The town lies at an altitude of about 1850 m above sea level. The main rainy season extends from June to September, with an average rainfall of about 800 mm. The mean annual minimum and maximum temperatures are 12.3°C and 27.7°C, respectively, with an overall average temperature of 18.7°C. The mean relative humidity is 61.3% [13].

### 2.2. Study Design and Study Population

A cross-sectional study was conducted to determine the occurrence of nematode infections and associated risk factors in commercial poultry farms using the coprological technique. Based on the willingness of the farm owners, those farms officially registered in the study were purposively selected, and pooled fresh fecal samples were roundly collected from 60 poultry farms. The information regarding production types, chicken age, flock size, hygiene practices, footbath usage, and proximity to other farms was recorded using a structured format through a short interview with the owners. The study farms were grouped into three production types (broiler, layer, and dual-purpose), and age was categorized as either young or adult according to the methodology used by Woldemariam and Wossene [14]. Farms were grouped by scale as small (50–500 birds), medium (501–1000 birds), and large (1001–10,000 birds) as described by Sime [15].

### 2.3. Sample Collection

Representative fresh fecal droppings were collected from different locations throughout the poultry farm and pooled into a universal bottle. Each sample was labeled with the farm code and transported to the parasitology laboratory at Addis Ababa University, College of Veterinary Medicine and Agriculture, for the detection of GI nematodes. Laboratory procedures were performed immediately upon arrival.

### 2.4. Laboratory Analysis

Fecal samples were processed using a floatation technique with saturated sodium chloride following the procedure described by MAFF [16] and Christie et al. [17]. This method relies on the principle that the parasite eggs are less dense than the flotation fluid medium. Briefly, 3 g of fecal sample was placed into a beaker, and 42 mL of flotation fluid was added. After thoroughly mixing, the fecal suspension was sieved through a tea strainer into another beaker. Then, the fecal suspension was poured into test tubes until the meniscus formed, covered with a coverslip, and left to stand for 20 min. The coverslips were then removed, placed on a clean slide, and examined under a compound microscope at 10 × 10 magnification. Identification of parasites was done based on the morphology of eggs, as described by Soulsby [18].

### 2.5. Data Management and Analysis

The collected data were entered in Microsoft Excel (MS Excel, 2016), and analyses were done using R statistical software (Version 4.1.2). Descriptive statistics were used to summarize the findings, and the Pearson chi-square test was applied to assess the association between each risk factor and the occurrence of nematode infections. Statistical significance was determined at 95% confidence interval and 5 degrees of freedom. A *p* value of less than 0.05 is considered to be statistically significant.

## 3. Results

**Figure 2:**
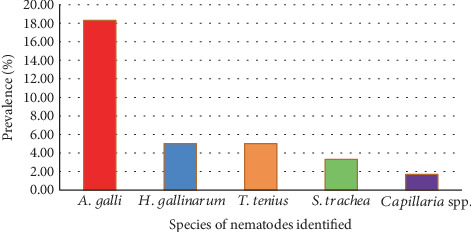
Percentage of GI nematodes that infected farms in Bishoftu.

In this study, out of the 60 fecal samples obtained from poultry farms, 19 (31.7%) tested positive for eggs of one or more nematode parasites. Five major nematode parasite species identified were as follows: *A. galli* 11 (18.3%), *H. gallinarum* 3 (5%), *T. tenuis* 3 (5%), *S. trachea* 2 (3.3%), and *Capillaria* species 1 (1.7%) (Figure 2).

In this study, poultry farm biosecurity and management practices were assessed to evaluate their association with the occurrence of nematode infections. Of the 39 farms using wet cleaning, 3 (7.7%) tested positive for nematode parasites, which was a significantly lower (*p* < 0.05) infection rate than the farm that did not use wet cleaning (16.2%). The farms using chemical cleaning also had a significantly lower (*p* < 0.05) infection rate (7.7%) as compared to those not using chemical cleaning (12.7%). Thus, the incidence of nematode infection was significantly reduced (*p* < 0.05) by wet cleaning and chemical cleaning. Furthermore, 17 (62.96%) of the 27 farms without footbaths tested positive for GI nematode infection, which was significantly higher (*p* < 0.05) than the farms using footbaths (6.06%) (Table 1). When comparing the infection rates among the production types, out of 11 broiler farms examined, none of them tested positive for nematode parasite eggs, one of two (50%) dual-purpose chickens, and 18 (38.1%) of the 47 investigated layer chickens tested positive for the GI nematode infection. The difference in infection rate between these production types was statistically significant (*p* < 0.05) (Table 1).

The distance between the sampled farm and other farms was one of the risk factors that had significance (*p* < 0.05) on GI nematode infection. Farms located very near to others had a high GI nematode infection rate, which was 77.8%. Age group and farm flock size did not demonstrate a significant difference (*p* > 0.05) on GI nematode infection. Six (22.2%) of the 27 young farms were tested positive for GI nematode parasites, whereas 13 (39.4%) out of the 33 farms with adult chickens were tested positive (Table 1).

## 4. Discussion

In this cross-sectional study, 19 out of 60 poultry farms (31.7%) were found to be positive for nematode parasite infections. This finding was comparable with a previous study conducted in Jimma town, Southwest Ethiopia, by Yadeta et al. [9], who reported a 36.97% prevalence of GI nematodes in chickens. However, the prevalence observed in the current study is lower than previously reported by Berhe et al. [5] in Mekelle town (90.60%), Beyene et al. [10] in and around Bahir Dar town (46.9%), Temesgen et al. [19] in Ambo, Ethiopia (60.0%), and by Matur et al. [20] in Nigeria (53%). The differences in GI nematode prevalence among these studies may be attributed to chicken management systems, sample size, study methods, and parasite control practices employed across different locations. The relatively lower prevalence found in the present study could be related to the management system. The samples were collected from intensively reared commercial poultry farms, whereas most of the previously mentioned studies involved village chickens, which are raised under an extensive or semi-intensive management system. Such management systems are more likely to be exposed to parasitic infections due to scavenging behavior and high possibility of contact with contaminated environments. This difference in exposure risk could account for the lower prevalence observed in intensively managed systems.

In contrast, the present prevalence of GI nematode infection finding was higher than the reports of GI parasites by Afolabi et al. [21] in Nigeria (20.5%) and Baboolal et al. [22], who reported a 10.5% prevalence of helminth infection in broiler chickens in Trinidad, an island. The difference in nematode prevalence between this and other studies may be because of differences in geographical location, chicken breed, and other factors. Rahman et al. [23] have noted that nematode infection rates are influenced by multiple factors such as rainfall patterns, soil type, locality, management systems, and the types of food given to the chickens, all of which can vary significantly by location. Furthermore, the intensive management of the poultry farm used in the present study ensured that the flock received better care in terms of biosecurity, hygiene, food, suitable preventative medical programs, and general management as compared to local and scavenging chickens.

The most prevalent nematode species identified in the present study was *A. galli* (18.3%), followed by *H. gallinarum* (5%), *T. tenuis* (5%), *S. trachea* (3.3%), and *Capillaria* species (1.7%). A similar finding by Ara et al. [24] in Kashmir, India, and Temesgen et al. [19] in and around Ambo, Ethiopia, reported that *A. galli* was the most abundant nematode species encountered in the study, followed by *H. gallinarum* and *Capillaria* spp. However, a contrasting report by Khan et al. [25] in Pakistan indicated that *H. gallinarum* is the most frequent nematode (38.89%) and is followed by *A. galli* (27.7%).

In the present study, *A. galli* was the more prevalent parasite (18.3%), which was comparable with the finding by Alemu [26] in Debre Zeit Agricultural Research Center Poultry Farm (20.79%), and Junaidu et al. [27] in Giwa Local Government, Nigeria (17.0%). However, the prevalence of *A. galli* (18.3%) in this study was much lower than that previously reported by Ashenafi and Eshetu [12] in central Ethiopia (55.26%) and Tesfaheywet et al. [28] in Haromaya (38.0%), Berhe et al. [5] in Mekelle (68.8), and Ara et al. [24] in India (34.25%). These differences in prevalence of GI nematodes might be attributed to the variation in management systems, deworming practices, sample sources (intensive vs. extensive production), and/or agro-ecological conditions in the study area.

Among the different age groups, adult chickens showed a higher prevalence (39.4%) of GI nematode parasitic infection compared to the younger chickens (22.2%). This finding is consistent with the finding of Beyene et al. [10], who reported a higher prevalence in adult chickens (50.6%) than in the younger groups (38.9%). Similarly, Tesfaheywet et al. [28] in Southeastern Ethiopia found that helminthiasis was more common in adults (45.9%) than in young chicks (38%). In contrast, Temesgen et al. [19] observed a higher prevalence of GI nematodes in young chickens than in adults in Ambo, Ethiopia. Likewise, Sarba et al. [29] in West Shoa Zone Central, Ethiopia, reported that GI nematode is more prevalent in young (90.2%) than in adult chickens (75.3%).

Even though young chickens are more susceptible to parasitic infection due to their underdeveloped immune system, the lower prevalence observed in this finding might be a farm management practice. In commercial poultry farm systems, farmers may use anthelmintics more frequently in younger chickens to protect the chickens from early parasite burden, because drug residues at this stage are not their concern. On the other hand, adult chickens may have had longer exposure to infective larval stages contributing to a higher cumulative infection rate. These factors may explain the high prevalence observed in adult chickens in this study.

In this study, flocks raised for meat production (broiler) had a lower prevalence of GI nematode infections compared to those raised for egg production (layers). Similarly, the study conducted by Afolabi et al. [21] in Nigeria reported a significantly lower prevalence of GI nematodes in broilers than in layers. However, warm temperatures (20°C–30°C) and humid (> 70% RH) conditions in broiler houses do favor nematode survival and transmission, but this difference may be attributed to the fact that the lifespan of broiler chickens is very short compared to the layer breed, so the probability of getting the parasitic infection could be higher as they stay on the farm for a long period of time [30, 31].

In this study, the use of footbaths, along with regular cleaning and disinfection practices during downtime, significantly (*p* < 0.05) decreased the prevalence of the GI nematode parasite infection as compared to the farms that did not apply wet cleaning and chemical cleaning of poultry houses during downtime. As described in different investigations such as the Review on Diseases and Health Management of Poultry and Swine by Serbessa et al. [32] and the report by Mekuria and Bayessa [33], proper wet cleaning with detergents and chemical disinfection plays a role in destroying different types of nematode parasites from poultry farms. Biosecurity practices such as appropriate distance between poultry farms have also played a role in the prevalence of the GI nematode parasitic infection. Very near distances from other poultry farms were significantly (*p* < 0.05) higher in prevalence as compared to those farms located far from other poultry farms. This finding indicates that good biosecurity practices, including appropriate distance between poultry farms, are very important in the minimization of nematode disease occurrence.

## 5. Conclusion and Recommendations

This study has demonstrated five types of GI nematode parasites in chickens in intensive management systems in the study area within the study period. The findings showed that risk factors such as farm cleaning practices, type of production, and farm location have influenced the occurrence of poultry nematode parasites. Therefore, poultry farm owners should keep litter dry and clean to prevent the development of GI nematode eggs; follow all-in/all-out production with thorough cleaning and disinfection between flocks; implement deworming based on fecal egg counts and veterinary advice; enforce strong biosecurity measures, including limiting visitors and using footbaths; and educate farm staff on parasite control and farm management practices. Additionally, further research on the epidemiology of the diseases in different types of poultry production systems should be conducted.

## Figures and Tables

**Table 1:** Prevalence of gastrointestinal nematode infection by associated risk factors.

**Risk factors**	**Category**	**No. of farm sampled**	**No. of positive**	**Prevalence (%)**	**Chi-square**(**χ**^2^)	**p** **value**
Footbath	Yes	33	2	6.06	22.22	< 0.05⁣^∗^
	No	27	17	62.96		

Chemical cleaning	Yes	42	7	16.7	14.56	0.001⁣^∗^
	No	18	12	66.7		

Wet cleaning	Yes	39	3	7.7	29.60	< 0.05⁣^∗^
	No	21	16	76.2		

Breed	Broiler	11	0	0	6.36	0.04⁣^∗^
	Dual	2	1	50		
	Layer	47	18	38.3		

Flock size	Large	40	11	27.5	2.43	0.3
	Medium	7	4	57.14		
	Small	13	4	30.8		

Age	Young	27	6	22.2	2.02	0.2
	Adult	33	13	39.4		

Distance from another farm	Very near	9	7	77.8	10.41	< 0.05⁣^∗^
	Near	43	10	2.3		
	Far	8	2	25		

⁣^∗^A *p* value < 0.05 indicates a statistically significant association between the risk factor and nematode infection.

## Data Availability

The data that support the findings of this study are available from the corresponding author upon reasonable request.
